# The Potential Wind Power Resource in Australia: A New Perspective

**DOI:** 10.1371/journal.pone.0099608

**Published:** 2014-07-02

**Authors:** Willow Hallgren, Udaya Bhaskar Gunturu, Adam Schlosser

**Affiliations:** The MIT Joint Program on the Science and Policy of Global Change, Massachusetts Institute of Technology, Cambridge, Massachusetts, United States of America; CNRS, France

## Abstract

Australia’s wind resource is considered to be very good, and the utilization of this renewable energy resource is increasing rapidly: wind power installed capacity increased by 35% from 2006 to 2011 and is predicted to account for over 12% of Australia’s electricity generation in 2030. Due to this growth in the utilization of the wind resource and the increasing importance of wind power in Australia’s energy mix, this study sets out to analyze and interpret the nature of Australia’s wind resources using robust metrics of the abundance, variability and intermittency of wind power density, and analyzes the variation of these characteristics with current and potential wind turbine hub heights. We also assess the extent to which wind intermittency, on hourly or greater timescales, can potentially be mitigated by the aggregation of geographically dispersed wind farms, and in so doing, lessen the severe impact on wind power economic viability of long lulls in wind and power generated. Our results suggest that over much of Australia, areas that have high wind intermittency coincide with large expanses in which the aggregation of turbine output does not mitigate variability. These areas are also geographically remote, some are disconnected from the east coast’s electricity grid and large population centers, which are factors that could decrease the potential economic viability of wind farms in these locations. However, on the eastern seaboard, even though the wind resource is weaker, it is less variable, much closer to large population centers, and there exists more potential to mitigate it’s intermittency through aggregation. This study forms a necessary precursor to the analysis of the impact of large-scale circulations and oscillations on the wind resource at the mesoscale.

## Introduction

The general climatology of the winds in Australia has been documented on a national basis [1], [2], [3] and at the state level [4], [5], [6], [7], using a variety of methodologies [8]. Such climatologies indicate that Australia has wind resources that are in places comparable to those in northern Europe, and indicate that the location of the strongest winds is in western, southwestern, and southern Australia, and southeastern coastal regions [8].

The physical quantity conventionally used to describe the wind energy potential in Australia is wind speed in m/s, whereas in the USA, wind atlases show maps of wind power density (WPD) to describe the quality of the wind resource. Most previous published studies use the mean to characterize the central tendency of the wind resource, however histograms of the wind resource measured using wind power density are characteristically skewed with long-tailed distributions [9] (Figure S1). Therefore, wind power studies based only on the total mean WPD do not give a representative picture of the central tendency of the wind power potential and also omit valuable information in terms of wind intermittency, variability and the temporal distribution of power generation [10], which would affect estimates of power production and required backup [11].

Variability in the wind resource has major ramifications for the economics and therefore the feasibility of wind power generation and distribution, and hence measures of variability are useful for wind energy policy makers. Yet, very few atlases show maps of wind variability [9], and when they do it is typically in terms of the standard deviation of the wind speed or WPD. However, the economic viability of wind power as an alternative energy source strongly depends on how reliable the resource is, in terms of its availability and persistence, as well as other factors such as proximity to high-capacity power transmission lines, and how remote it is from population centers and the electricity grid. The reliability of wind power can in theory be increased by mitigating the natural intermittency of the wind resource, by aggregating power from wind farms that are geographically dispersed, with the aim of achieving a more continuous wind resource over large areas, and there have been several studies trying to address this issue [12], [13].

Wind power production doubled in the 5 years to 2012, and has grown 340% since 1997, to meet 3.4% of Australia’s total electricity demand and 26% of total renewable energy generated, which is a bit less than half that generated by hydropower [14]. Wind power is likely to become economically competitive in the coming decades, and is projected to grow by 350% when wind power projects currently in development come online in the next few years [15]. This projected expansion of wind energy conforms to national policies that were designed to lower carbon emissions, including legislation that was introduced to put a price on carbon, and the Renewable Energy Target of 20% by 2020 [15]. In light of this policy directive, there is a need to increase the accuracy and practical relevance of the assessment of Australia’s wind power resource.

We assess Australia’s potential wind power resource with alternative metrics of abundance, variability and intermittency that provide deeper insights about the stability of the wind resource at a widespread deployment scale [9], [11] over long time periods, using a robust, multi-decadal dataset.

Several authors explore the variability and intermittency of the wind resource at many scales [16], [17]. There are fewer studies at the mesoscale scale range than at smaller scale ranges, despite the fact that knowledge of variability at this scale is important to the management and control of wind power generation [16]. Our study focuses on variability and intermittency at the hourly scale and above -the mesoscale- and addresses the type of scenario, to take just one example, in which long wind lulls spanning weeks, during sustained periods of high pressure, have been known to occur in countries such as the UK and Germany (Oswald et al 2008 [18], telegraph article [19]). These instances have implications for the reliability of power generated, as well as the potential backup and storage required to sustain power delivery. The goal of the present paper is to characterize the wind resource in Australia and its inherent variability, as a necessary precursor to studies of the impact of large-scale climate oscillations on the variability of the wind resource at different scales.

Questions our study asks include: (1) What is the geographical distribution of the abundance, variability, availability, and persistence of wind power density (WPD), and do these differ with higher turbine hub heights? (2) Where can wind intermittency be mitigated by the aggregation of geographically dispersed wind farms?

## Methods

### 2.1. Data

We have sought to address some of the limitations of previous wind resource studies that used data that had a coarse spatial and temporal resolution, a relatively short record length, and sparse and uneven coverage [20], [11]. We used 31 years of hourly 1/2°×2/3° resolution MERRA (Modern Era Retrospective Analysis for Research and Applications [21] data (from 0030 on January 1st, 1979 to 2330 on 31st December, 2009) to reconstruct the wind field at several turbine hub heights 50 m, 80 m, and 150 m, since the MERRA dataset does not provide wind speeds at different hub heights. These heights were chosen to represent the recent 1990’s (US) 50 m standard wind turbine hub height [22], [23], and the 80 m hub height, which has become more common as technology develops, and the potentially much higher hub heights in the future.

Wind speed and then wind power density were computed at these different heights using boundary layer flux data (consisting of such parameters as surface roughness, displacement height and friction velocity) and similarity theory of the atmospheric boundary layer [9]. By doing this, we sought to improve on previous wind resource constructions that used a constant scaling exponent (irrespective of surface roughness) to scale the wind speed from a lower altitude (usually 10 m) to that of the turbine hub height. We use WPD (W m^−2^) to describe the wind resource as it is a function of not only wind speed but also density, which also varies in space and time. It indicates how much wind energy can be harvested at a location by a wind turbine but is independent of wind turbine characteristics. In a recent study, Farkas [24] found that non-consideration of air density causes an root mean square (RMS) error of 16% in wind potential, which is a considerable difference, and therefore air density should be an important consideration in estimating the wind resource potential. The domain considered for our study spans the entire Australian continent plus Tasmania, between 10°S and 45°S latitudes and 110°E and 155°E longitudes.

While the resolution of the data used in this study is lower than the mesoscale, there have also been many studies that establish the utility of data at the GCM resolution (e.g. Schwartz and George, 1999) [25] for understanding the variability and impact of large-scale circulations at a regional scale. Several studies have used a similar dataset, although with a shorter record length, to estimate the potential wind resource in China [26] and also globally [27]. However, for studying inter-decadal variability, we argue that the longer record length of the data is as essential an attribute. This is because, according to sampling theorem, a dataset has to have at least 20 years of data for understanding inter-decadal variability. Hence this construction was designed, and is most appropriate, for such studies.

All other constructions that span only a few years fail to represent such variability. Moreover, studies such as those by Pryor, Barthelmie and Schoof (2006) [28], Chadee and Clarke (2013) [29], use data with a lower resolution than that used here, to study similar issues (inter-annual variability of wind indices across Europe, large-scale wind energy potential of the Caribbean, etc.). This would indicate that our data resolution is suitable for the purpose of our research, and represents an improvement to the resolution of a number of prior studies [22], [26], [27], [28], [29], [30], [31], [32].

Since Gunturu and Schlosser (2012) [9] have already done a thorough evaluation of the lowest model layer wind speed data taken directly from MERRA, and since this study uses the same data, as it is a continuation of theirs, it is unnecessary to reproduce this validation of the MERRA data here. As the original MERRA wind data that this study employs is in the public domain, the description of the methodology will enable others to construct the wind power density dataset that is used in this study.

### 2.2. Comparison with Existing Wind Climatologies in Australia

Here, the wind resource is constructed for a hub height of 80 m, and was compared with a publicly available map of 80 m wind speed, since a publicly accessible wind power density map was not available. This was done in order to understand the ability of this constructed dataset to reproduce large-scale spatial features. This map was originally published by the Australian government and also used by various state governments [15]. We also use publicly available maps of the location of wind farms in South Australia and New South Wales to validate our wind resource construction [33], [34].

### 2.3. Wind resource metrics

The metrics we use in our study are wind abundance, variability and intermittency in the form of availability and persistence [9], [11]. Most previously published studies use the mean to characterize the central tendency of the wind resource. Since the mean is not a robust measure of the central tendency for distributions with long tails, we use the median, which is immune to the extreme values in the distribution, as a robust measure of the central tendency of the wind resource, and provides a better evaluation of it’s abundance. As Pryor and Barthelmie (2011) [20] point out “there is a need for accurate data pertaining to metrics of the wind climate beyond the central tendency, and trends in annual mean wind speeds have little bearing on the viability of wind energy.”

Instead of using the standard deviation to represent the variability of the wind resource, we argue that the variability of the wind resource is better captured in terms of the robust coefficient of variation (RCoV), since it is calculated using the median, which we argue is a more accurate representation of the wind power at a given site than the mean. We also use the Inter-quartile range (IQR) as a measure of the statistical dispersion, higher values of which can indicate the greater possibility of ‘swings’ of the WPD at a location, and therefore the amount of backup power that needs to be maintained.

In addition to these measures of variability, we also look at two measures of the intermittency of the wind - availability (or lack of) and persistence - since these are important indicators of intermittency, which is recognized as one of the key limitations to large-scale installation of wind power. We apply the reliability theory concept of availability to wind power, as a measure of the temporal distribution of the wind resource, and therefore of the reliability of a wind power generation system. We calculate the percentage of hours in our time series where WPD>200 W m^−2^, and use the inverse of this - unavailability - of non-useful WPD (i.e. proportion of hours where <200 W m^−2^), to characterize the geographic distribution of the reliability of the wind resource [11], and as one measure of intermittency. Our rationale for choosing the 200 W m^−2^ cutoff is the same as Gunturu and Schlosser (2011) [11], and incorporates a number of contributing arguments, which are detailed in Text S1. Mean episode length (i.e. number of hours of WPD above 200 W m^−2^) was calculated as a measure of the persistence of the WPD, which is important in the planning and development of a robust deployment strategy for harvesting wind power.

We use Gunturu and Schlosser’s (2011) [11] technique to analyze the potential value of aggregating the power generated by geographically dispersed wind farms in a roughly 1000×1000 km box (19×19 grid cells), in order to mitigate intermittency in the wind resource. Values of anticoincidence [35], and null-anticoincidence were calculated for each grid cell (see Fig. 5 in Gunturu and Schlosser, 2011) by converting the time series of WPD at each grid point into a binary sequence of 1 s and 0 s depending on if the WPD is greater or less than the 200 W m^−2^ we use as the cutoff useful for viable commercial generation. We base our analysis of anticoincidence on these binary sequences. Two grid points are said to be anticoincident when the hourly time series of WPD is greater than 200 W m^−2^ at one of the two points, but not both, for 50% of the total length of the time series. We also calculate the null-anticoincidence, which offers a somewhat more relaxed criterion. Null-anticoincidence refers to the number of grid points in a roughly 1000×1000 km area surrounding a central point which have usable wind power (>200 W m^−2^), when the central point does not, for at least 50% of the time when there’s no wind at the central point [11]. If the region within this analysis area shows higher values of anticoincidence then this means that there will be fewer coincident lulls in the wind resource across the region, and that aggregating power from geographically dispersed wind farms will be more likely to mitigate the intermittency of the wind resource across the region as a whole.

Our choice for using a box this size for the anticoincidence analysis was based on the fact we are looking at the wind resource at a regional scale, hence, this is the scale at which we studied anticoincidence: the mesoscale. For more information on the rationale for the box size, refer to the Supplementary Information. In terms of the temporal scale used in this study, while there have been several methods and technologies to mitigate intermittency at the operational scale, such intermittency for the grid operations occurs at micro-to-hundreds of seconds. But for the scale that this study pertains to, no methods or technologies have yet been developed to deal with intermittency, to the knowledge of the authors, at the scale of one hour or more, in which case, the issues of back up and resource adequacy become important.

## Results and Discussion

Wind speed and wind power density were computed at several wind turbine hub heights using boundary layer flux data from the Modern Era Retrospective-analysis for Research and Applications (MERRA) [21] and similarity theory of the atmospheric boundary layer [9]. We use wind speed to compare our results to existing wind atlases (as the reference atlas for Australia uses wind speed instead of wind power density to measure wind power potential), as well as a range of metrics to analyze wind power density, including wind abundance, variability, and intermittency in the form of availability and persistence [11], [9]. Detailed descriptions of the data and methodology are described in the Methods section.

### 3.1. Comparison of MERRA and Australian Government maps of wind speed at 80 m

**Figure 1:**
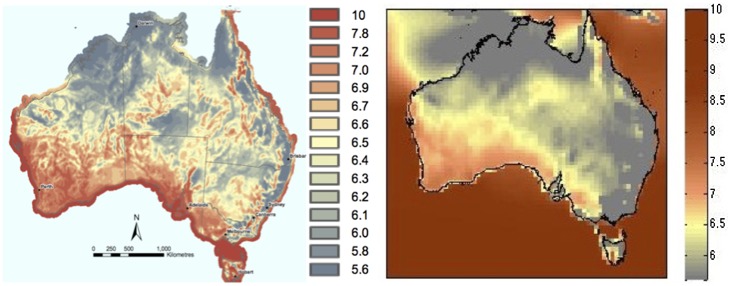
Comparison of mean wind speed (m/s) at an 80 m turbine hub height across Australia. (Left) Map developed by the Australian Government Department of the Environment, Water, Heritage and the Arts in 2008, and (right) the map constructed from MERRA data.

Our approximately 50 km×67 km (½ degree×⅔ degree) map of 80 m above ground level wind speed (Fig. 1) is quantitatively and geographically similar to the 9 km×9 km resolution map of wind speed at the same height produced by the Australian Government Department of the Environment, Water, Heritage and the Arts (hereafter referred to as AGD) [15]. This map was created by WindLab (www.windlab.com) for the AGD and is derived from observed weather station data taken from Bureau of Meteorology weather stations for the years 1995–2005, for the entire continent, and supplemented with commercially produced meteorological datasets, which are then assimilated into a high resolution broad-area wind mapping model called WindScape [36]. WindScape uses a regional scale weather model (The Air Pollution Model (TAPM [37]) to improve the resolution of the observed data, and also a fine scale computational fluid dynamics model Raptor and/or Raptor-NL to create fine scale resolution maps of the wind resource over broad areas. The maps created are validated and adjusted to achieve consistency with observational data at ground level [38].

While our construction of the wind resource matches qualitatively and quantitatively very well with that of the AGD map overall, there are differences between the two maps in some regions. Our results mostly show slightly lower values for most areas compared to corresponding areas on the AGD map (Table 1). For example, a comparison of our map with the maps of NSW [33] and South Australian [34] wind farms, indicates regions where areas of better wind resources, as shown on our constructed map, coincide with existing wind farm deployments on [33] and [34], particularly in NSW, even though wind speed values in these areas might be slightly lower on our constructed map, than on the AGD map, as shown in Fig. 1. On that map, this is not always the case - it shows even better wind resources outside of these regions of wind farm deployment. This indicates that our map does actually capture areas of good wind resource in areas where there are existing wind farms.

**Table 1:** Comparison of the range of values (m/s) in many areas of the 80 m wind speed map constructed from MERRA data to the one produced by the Australian Government.

Regions of similarity	MERRA data map	Australian government map
East coast, Tasmania	5.6–7.0	6.5–7.8
Western Victoria	6.5–7.0	Mostly >7.0
SE South Australia	6.4–7.2	Up to 7.8
Central Australia	5.6–7.0	5.8–6.6

The first region encompasses much of the East coast, and includes southeast and northeast QLD, and Tasmania.

Furthermore, our coarser-resolution map of the wind resource shows fewer orographic effects of the Great Dividing Range than the AGD map. Nevertheless, our map captures precisely the areas where there are existing wind farms on the New South Wales Southern Highlands and Blue Mountains [33], [34]. So although our map has a resolution which does not capture as much topographical detail as the government map, it captures precisely the areas where there are existing wind farms, for instance, our map shows two small regions on the eastern seaboard of good wind resource, which is where all but one of the existing wind farms are currently located.

Reasons for the differences seen in these two maps could be due to the lower spatial resolution of our constructed map and the lower temporal record length of the AGD map. Since the AGD wind resource map has been constructed by running a mesoscale model (TAPM) for 11 years (and all other constructions also span only a few years), the record length of the construction is short compared to the record length of our construction, which represents an average over 31 years, that includes many years of low and high wind. Short record lengths do not represent interannual variability and climate scale (i.e. more than a few years) oscillations like the El Nino Southern Oscillation (ENSO) robustly.

### 3.2. Measures of abundance and variability

**Figure 2:**
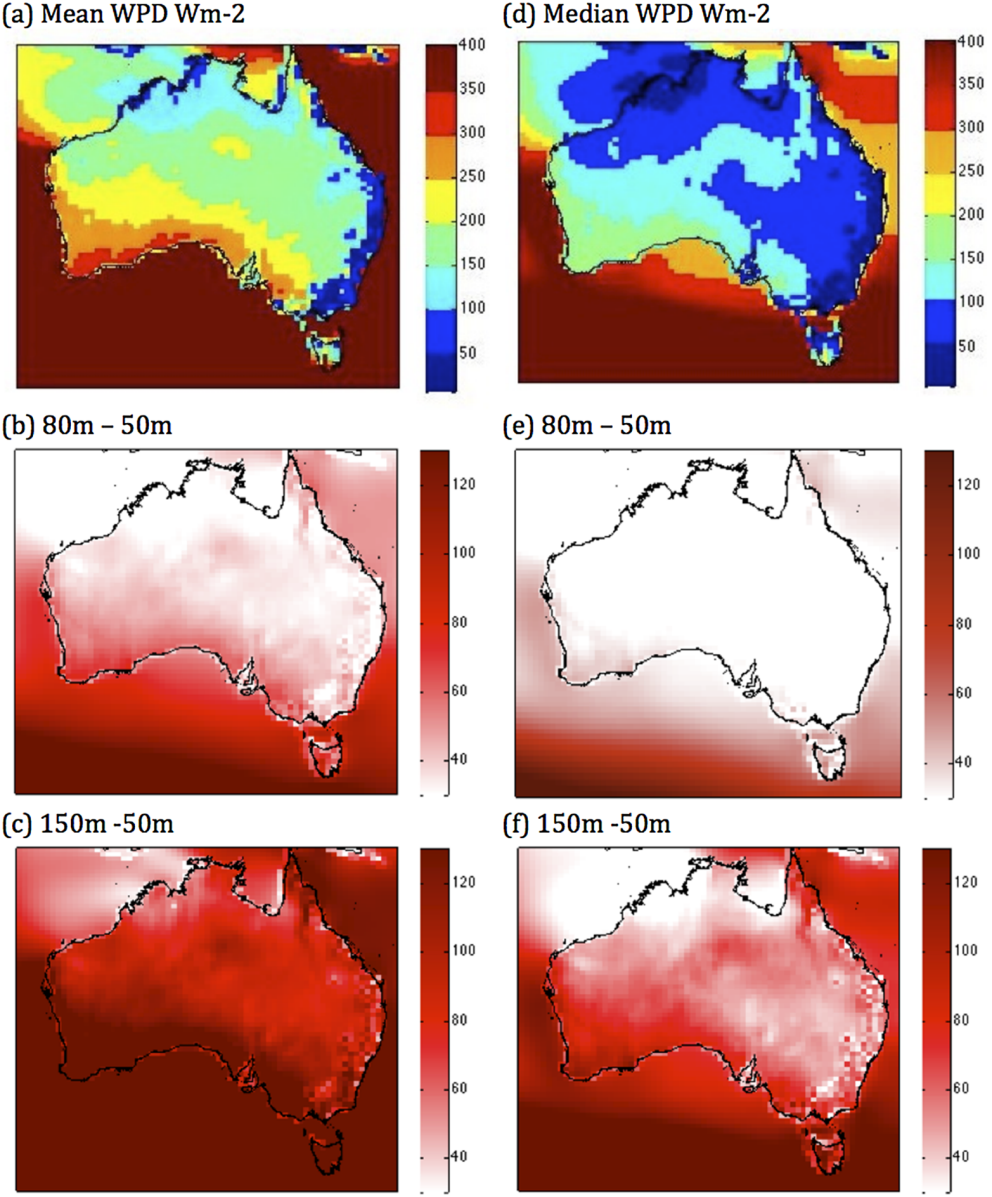
Measures of abundance. (a) The mean WPD at 50 m, (b) the change in the mean from 50 m to 80 m and from (c) 50 m to 150 m, (d) median wind power density at 50 m, (e) the change in the median from 50 m to 80 m and from (f) 50 m to 150 m. All units are W m^−2^.

Reflecting the wind speed patterns of previous Australian wind atlases, our constructed map of mean WPD at 50 m (Fig. 2a) shows that the strongest wind resources occur in southwest Western Australia, southern South Australia, and Tasmania, and south-western Victoria. It is lowest in mountainous areas along the Great Dividing Range in eastern Australia, in northwest Australia, and northwest QLD. Most of the continent has mean WPD values below 300, and most of the populated east coast of the country has values below 200 W m^−2^ at this resolution, which is the cutoff for the production of usable power that turbines can produce, the rationale for which is detailed in Text S1. As turbine hub height increases to 80 (Fig. 2b) and 150 m (Fig. 2c), there is an increase in mean WPD of up to about 40 and 100 W m^−2^ in the northern two-thirds of Australia and 80 and 160 W m^−2^ (and higher in Tasmania) in the south respectively. While the mean WPD construction reflects the other known datasets that illustrate wind speed, we extend the analysis that has historically been done, and look at other metrics of the resource that could be useful for assessing the economics of wind power generation and also for operational stability.

The map of median WPD at 50 m (Fig. 2d) indicates that a greater part of the continent has WPD below the 200 W m^−2^ value. Compared with the mean WPD in Fig. 2a, the median values are almost half of the mean values throughout much of the country. This implies that the distribution is very skewed, and hence we argue that the median is a much more robust measure of central tendency and therefore a more appropriate metric to represent WPD. As turbine hub height increases to 80 m (Fig. 2e) and then 150 m (Fig. 2f), there is less of an increase in median WPD compared to mean; up to about 30 and 80 W m^−2^ in the northern half of Australia, and up to about 50 and 120 W m^−2^ (and higher in Tasmania) along the southern part of the country. This scenario implies that the number of hours which show an increase in WPD are about the same as those which show a decrease, however the increase of WPD in those hours which show an increase, is greater than the decrease of WPD in the hours which show a decrease. We infer from this that variability and intermittency of the resource are increasing while the median resource is increasing.

**Figure 3:**
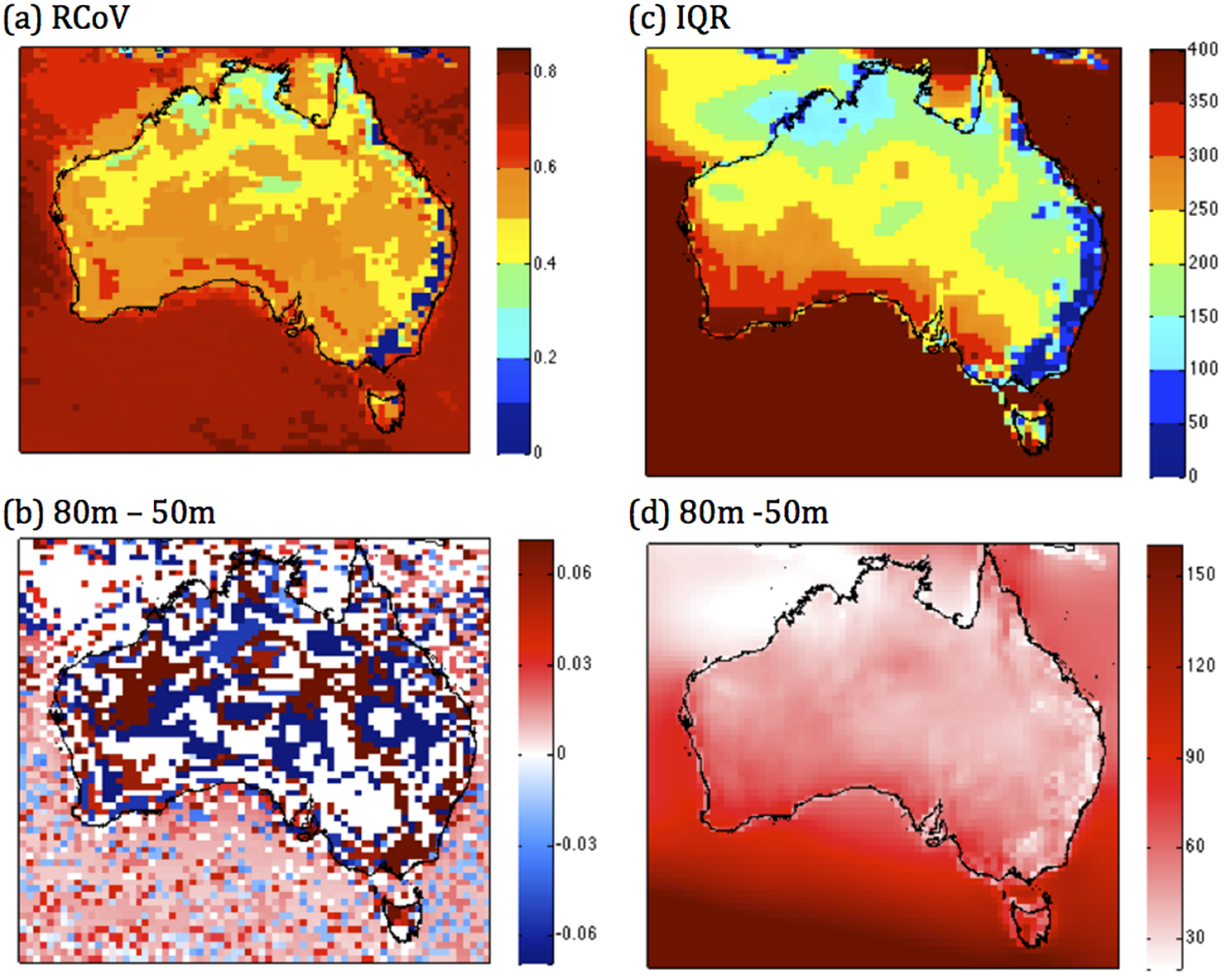
Measures of variation. (a) The robust coefficient of variation (RCoV -unitless) of WPD at 50 m, (b) the change in the RCoV from 50 m to 80 m, (c) inter-quartile range (IQR, W m^−2^) at 50 m, (d) the change in the IQR from 50 m to 80 m.

Most maps of the variability of the wind resource use the standard deviation. We do not use the normal standard deviation. In line with our argument that the median is a better metric, being non-parametric, we use the ‘robust coefficient of variation’ (RCoV) that is the ratio of median deviation about the median to the median. Our results show that the highest RCoV values occur in southwest Tasmania and WA, and in southern South Australia, but inland from the coastline, which indicates these areas have relatively higher variability compared to the abundance in terms of the median (Fig. 3a). The lowest values, indicating a less variable, more reliable wind resource, occur along the southeastern seaboard and in parts of northern Australia near the coast.

RCoV increases with hub height in some areas (e.g. southeastern Australia), and decreases in others (much of inland SA) (Figs. 3b, S2 (a)). This is because although the median increases with height everywhere, variability decreases in some regions and increases in others. When the median increases with the hub height, and the variability also increases as much or more, RCoV (which is the ratio of deviation to central tendency) also increases. The RCoV decreases when the median increases but the variation does not increase so much (i.e. the ratio decreases). A scenario where RCoV decreases with height indicates that raising the hub height would better harvest the greater wind resources at higher hub heights, with lowered variability and intermittency. With greater surface friction, the standard deviation of the wind in the boundary layer increases [39]. Therefore, the boundary layer roughness predominantly determines the impact that raising the hub height has on the RCoV of the wind resource.

The interquartile range (Fig. 3c) is a measure of an important measure of dispersion in the wind resource since it is immune from the effect of outlying extreme values. Thus it is one of the robust measures of dispersion. As such, it can provide an insight as to the possibility of swings in the wind resource and therefore the amount of backup power that needs to be maintained. At 50 m, the areas that show high IQR (Fig. 3c) tend to coincide with areas that have the highest mean and median WPD (Figs. 2a and 2d, southwest and southern parts of the continent), and increases more with turbine hub height in these areas (Fig. 3d, S2 (b)). The regions that have low mean WPD also have the lowest IQR (e.g. east coast). IQR increases with turbine hub height across the country (Fig. 3d, S2 (b)).

If we consider just abundance and variability, regions that have high WPD and low variability (as shown by IQR) are areas where the wind resource could potentially be harnessed economically. Unfortunately, in Australia, our analysis indicates that at the resolution of this study, the areas which have mean WPD>200 W m^−2^ also have an IQR of at least the same magnitude if not greater, though undoubtedly there are isolated areas where this would not be the case – but our relatively coarse dataset is unable to show this. However, an additional, very important consideration for harnessing wind power economically at a widespread deployment scale is the extent of its episodic nature - or intermittency.

### 3.3. Measures of intermittency and the potential for its mitigation

**Figure 4:**
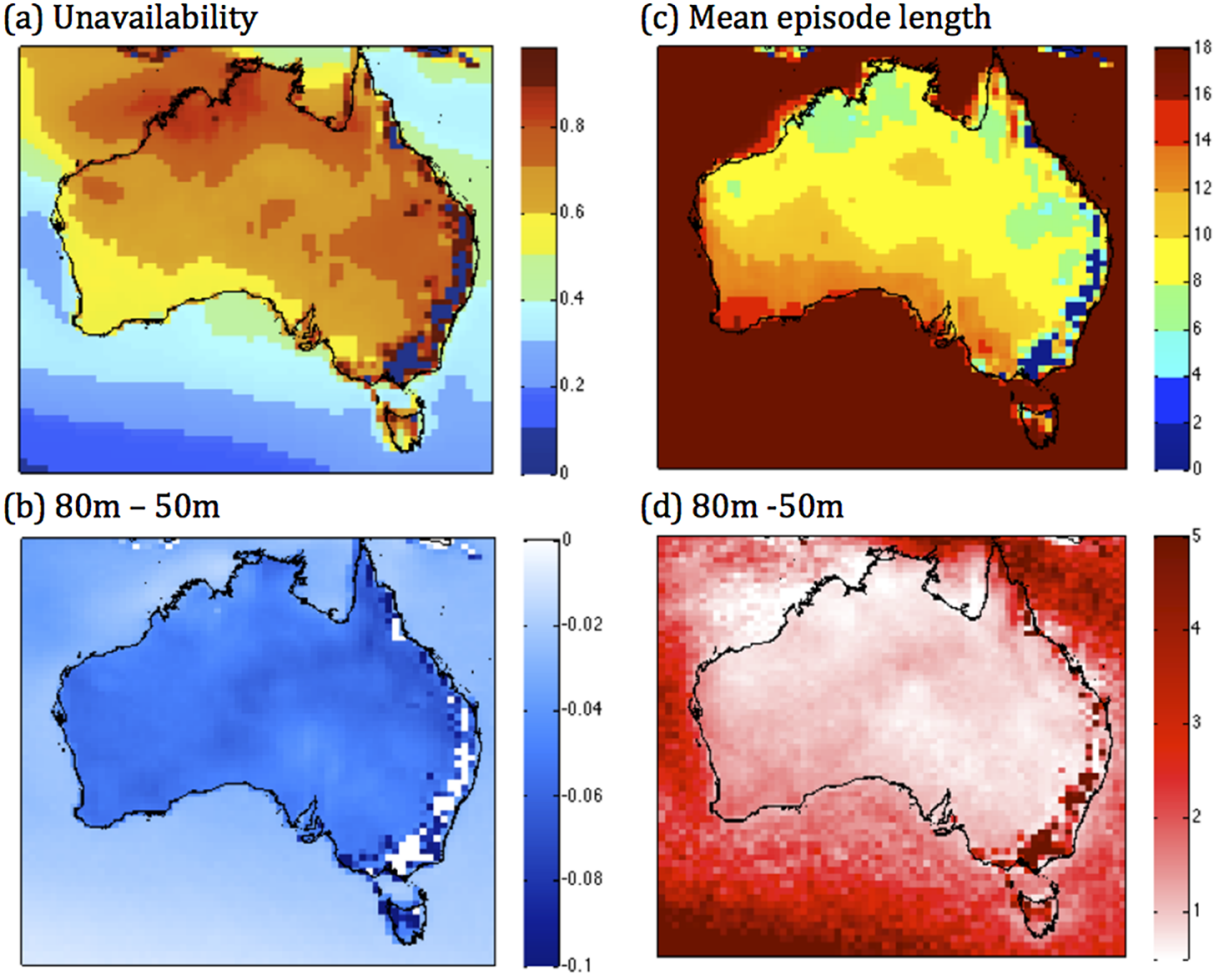
Measures of intermittency. (a) The unavailability of WPD at 50 m (fraction of time), (b) the change in the unavailability from 50 m to 80 m, (c) the mean episode length at 50 m (hours) (d) the change in the mean episode length from 50 m to 80 m.

To explicitly gauge the intermittency of WPD, we first consider a metric of unavailability (given as fraction of time WPD is less than a minimum threshold - see Data section). We find that unavailability, which decreases with height, is generally highest in the areas where mean (or median) WPD is low (far northwest Australia, northern Tasmania, and just west of the Great Dividing Range on the eastern seaboard). The lowest values are seen along the eastern seaboard, indicating more reliable winds in these areas. Large areas scattered throughout northern and eastern Australia exhibit relatively high values (above 0.65), with the southwestern third of the country exhibiting moderate values (Fig. 4a). Unavailability decreases with height, as might be expected (WPD increases, so given the 200 W m^−2^ threshold of availability, it also increases), except for the areas which have the lowest mean WPD values – higher altitude areas along the eastern seaboard - which show a negligible change in unavailability with a change in height (Figs. 4b, S3 (a)).

**Figure 5:**
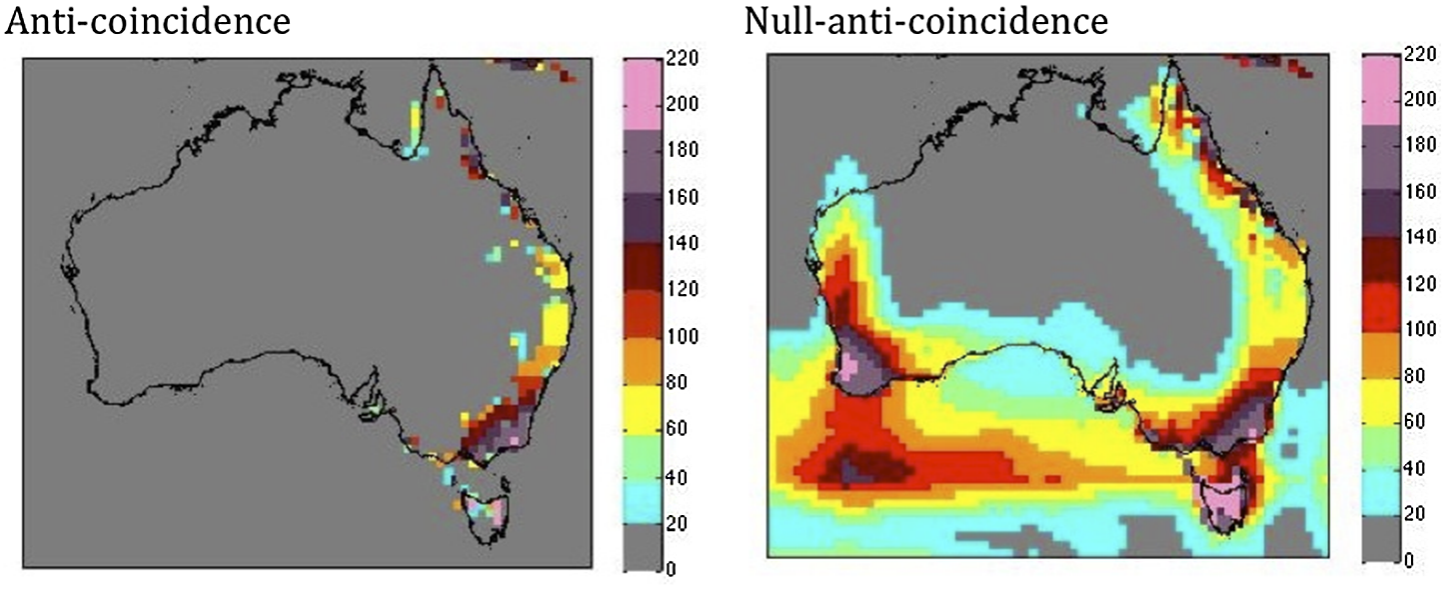
Anticoincidence (left) and Null-anticoincidence (right) of wind power density, at 50 m. Units indicate the number of grid points in a ∼1000×1000 km box surrounding the gridpoint in question which are anticoincident to the central gridpoint, which is when the hourly time series of WPD is greater than 200 W m-2 at one of the two points, but not both, for 50% of the total length of the time series.

The availability of WPD as a continuous resource over time is also considered. The spatial pattern of mean episode length (defined as the average time that WPD is continuously above the same threshold) closely resembles that of the mean WPD. We found that the mean episode length at 50 m hub height (c) is lowest in parts of the Great Dividing Range in the east of the country, where WPD is low, and highest in the southern Australia, south-west Western Australia, and Tasmania, where WPD is highest. Mean episode length increases with height most where the mean WPD is lowest, along the Great dividing range in the east (Figs 4d, S3 (b)). Conversely, areas where mean episode length is highest show only small increases (<2 hours) with increasing hub height to 80 m (Fig. 4d), and raising the hub height to 150 m results in a near linear response in terms of additional episode length (Fig. S3(b)).

The coincidence (or lack thereof - see Methods) of intermittent wind power in different places sets the scope of installed backup generation capacity required to maintain a steady power supply, as well as the benefits of the aggregation of wind resources. The areas with the lowest unavailability (suggesting low wind intermittency, or more reliable, steady winds) coincide with areas of moderate to high anticoincidence at 50 m, such as along the eastern seaboard. ‘Anticoincidence’ denotes the occurrence of one event without the simultaneous occurrence of another [35]. The greatest intensity of anticoincident points is in the southeast of the continent, including northeast Tasmania (Fig. 5). However, these areas also have a small episode length (suggesting less persistent winds), which suggests that the aggregation of wind farms may indeed help mitigate wind intermittency in the more densely populated southeast of Australia.

Davy and Coppin (2003) [40] found that the variability in the total wind power output in south east Australia can be reduced to some extent by wider distribution of numerous wind farms, but remains substantial, thus their analysis suggests some degree of anticoincidence of southeastern Australia’s wind resource. Their analysis spanned 4 years from March 1999 to March 2003, and also used hourly automatic weather station data from nine sites located on the SE Australian coast. It is useful to note that this period includes a marked La Nina episode and so the wind record may contain some anomalies, and may not be suitable for more general inferences. The record length used in our study, by contrast, is much longer and spans many ENSO cycles, and therefore can be used to infer the mean picture more robustly.

There are areas in Australia with relatively high intermittency - high unavailability and quite low mean episode length - such as northern and northwest Australia, that overlap a vast swathe of the continent west of the Great Dividing Range that shows little anticoincidence of WPD. These are the areas where aggregating turbines would be least effective, at the spatial and temporal scales analyzed.

However, an analysis of the null-anticoincidence (Figure 5) across Australia suggests that there may be some merit in linking wind farms across large areas to increase the reliability of the power supply in areas which show low anticoincidence and moderate to high intermittency, such as parts of the northern QLD coast, inland NSW, and parts of western Victoria and Tasmania, all of which show high values of null-anticoincidence. This may improve the reliability of wind power in these areas. These results agree well with previous research that has shown the coexistence of higher values of anticoincidence with regions that have high topographical inhomogeneity (i.e. mountain ranges) and proximity to the sea. This research has also co-located low anticoincidence areas to low surface roughness (flat terrain), semi-arid climate and terrains, with climate characterized by anti-cyclones which occur over large areas, leading to a large coincidence of low wind states across these high pressure systems [11].

## Summary and Conclusions

Our study suggests that many areas with the strongest widespread wind resource, in terms of both mean and median WPD (SW Western Australia, southern South Australia and Tasmania, and SW Victoria) also score relatively highly on measures of variability (IQR, RCoV) and exhibit moderate levels of intermittency, in terms of reliability (i.e. unavailability) and persistence (mean episode length). Much of the areas which have moderate to high wind intermittency also have very low anticoincidence, as defined in the Methods section, suggesting that there are large expanses of the continent in which aggregating turbines would be less effective, based on our study, at the spatial and temporal scales analyzed (keeping in mind the limitations of this study, described below). These areas also tend to be geographically remote from the bulk of the Australian population on the east coast (certainly in Western Australia, Northern Territory and South Australia), disconnected from the east coast’s electricity grid (Western Australia, Northern Territory), and often are not connected or located near enough high capacity electricity infrastructure (parts of South Australia) [41], all of which would decrease the potential economic viability of wind farms in these locations.

However, in eastern Australia (along the Great Dividing Range and the eastern seaboard), many areas exhibit a comparatively poorer wind resource (in terms of the mean and median), and the broad scale mean WPD is below the 200 W m^−2^ cutoff. However, the variability is also lower in these areas, the reliability is better, and the potential to mitigate intermittency (in the form of relatively low persistence) by the aggregation of wind farms, is larger; these areas tend to have higher values of anticoincidence, and null-anticoincidence. Our results broadly agree with those of Davy and Coppin (2003) [40] who demonstrated that variability in the total wind power output in south east Australia can be reduced to some extent by wider distribution of numerous wind farms.

There are several assumptions and limitations of our study which require articulating, the most important being the mapping scale issues that this study raises, whereby coarser resolution maps can overestimate the area available at a given wind speed, and will also potentially fail to depict many areas with good resources which occur at a scale smaller than the resolution our study employs (1/2×2/3 degree, or about 55×73 km square) [8]. Therefore, we acknowledge that our results are at least partly scale and resolution dependent. That being said, the continuous assimilation of observations to run the model enhances the efficacy of the MERRA data, i.e. if there are many sites that have good subgrid scale wind resources, this will be taken into consideration because the observations at these point locations are fed into the data assimilation cycle.

We assumed a neutral boundary layer, as do most of the wind resource assessments, including that by National Renewable Energy Laboratory (NREL) [22]. The wind energy atlas of the United States [22] justifies the neutral boundary condition as a first approximation, because the wind speeds (4–25 m/s) at which much of the power is produced in turbines occur at neutral stability. Parameterization of boundary layer stability into wind resource estimation is still a much researched area and we are working towards one such improvement.

The temporal resolution of the MERRA dataset is one hour, and as such, sub-hourly wind intermittency cannot be studied, even though this type of shorter scale intermittency can impact the voltage and frequency stability of a power grid [11]. Also, the MERRA data is created from the assimilation of observational data and satellite remote sensed data into a global model, and will reflect any imperfections of the model and the assimilation procedure, and will have an influence on the results presented here.

These limitations notwithstanding, we note that our data and results are not meant to be used for assessments of the deployability of wind farms at individual sites. Our wind resource construction is a tool to understand the geophysical nature of the resource at a regional scale and its variability, and the impact of large-scale atmospheric circulations and phenomena on the resource and its variability.

For this purpose, the multi-decade span of the MERRA data provides a more robust assessment of the temporal characteristics (i.e. mean, median, availability, intermittency, etc.) of wind power than that used in other studies. As described previously, while the data sets that exist have high spatial and temporal resolution, they do not have the record length required to assess the variability of the resource at the regional scale over longer time scales.

On the other hand, the constructed wind resource data described here uses a much longer record length, and this will allow future studies to utilize it to analyze the variability of the resource at different time scales (like the intra-seasonal and ENSO cycle time scales) and in response to different atmospheric oscillations like the El Nino Southern Oscillation and the Madden Julian Oscillation. This data will also be useful for analyzing the economic viability and the levelized costs of wind power compared to other energy sources, as well as for developing strategies for deployment such as the best pattern for aggregation. Studies such as this can conceivably delineate how far intermittency can be mitigated by aggregation and could play a role in the faster deployment of wind farms.

## Supporting Information

Figure S1 **An example of a histogram of wind power density that shows a typical skewed distribution.**
(TIFF)

Figure S2 **Measures of variation.** (a) the change in the RCoV from 50 m to 150 m, (b) the change in the IQR from 50 m to 150 m.
(TIFF)

Figure S3 **Measures of intermittency.** (a) the change in the unavailability from 50 m to 150 m, (b) the change in the mean episode length from 50 m to 150 m.
(TIFF)

Text S1 **Rational for the cut-off employed to calculate the intermittency metrics.**
(PDF)
