# Supplementary figures and images for: The Potential Wind Power Resource in Australia: A New Perspective
Source: PLoS One. 2014 Jul 2;9(7):e99608. doi: 10.1371/journal.pone.0099608 (PMC4079217; doi:10.1371/journal.pone.0099608)

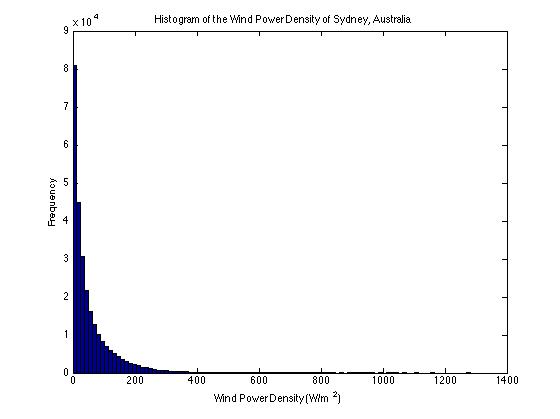

Supplement: Figure S1 — An example of a histogram of wind power density that shows a typical skewed distribution. (TIFF) [file pone.0099608.s001.tiff]

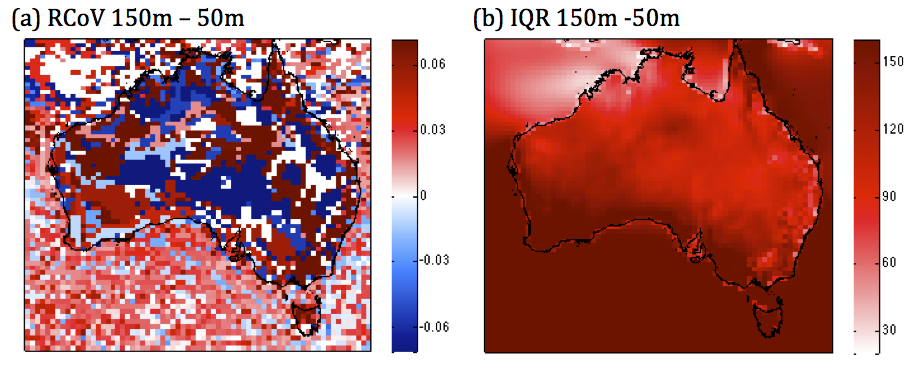

Supplement: Figure S2 — Measures of variation. (a) the change in the RCoV from 50 m to 150 m, (b) the change in the IQR from 50 m to 150 m. (TIFF) [file pone.0099608.s002.tiff]

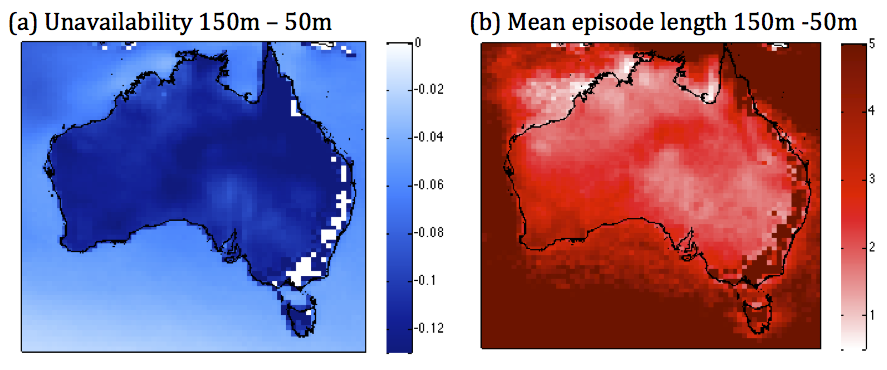

Supplement: Figure S3 — Measures of intermittency. (a) the change in the unavailability from 50 m to 150 m, (b) the change in the mean episode length from 50 m to 150 m. (TIFF) [file pone.0099608.s003.tiff]
