# Supplementary material for: The Potential Wind Power Resource in Australia: A New Perspective
Source: PLoS One. 2014 Jul 2;9(7):e99608. doi: 10.1371/journal.pone.0099608 (PMC4079217; doi:10.1371/journal.pone.0099608)
Supplement: Text S1 — Rational for the cut-off employed to calculate the intermittency metrics. (PDF) [file pone.0099608.s004.pdf]

Supporting Information

Rational for the cut-off employed to calculate the intermittency metrics

Our rationale for choosing the 200 W m^{-2} cut-off for the calculation of the intermittency metrics, is the same as Gunturu and Schlosser (2011), and incorporates a number of contributing arguments. The ReEDS (Renewable Energy Deployment System) model uses an annual mean WPD of 300 W m^{-2} to filter the sites for commercial scale power production. ReEDS is a model used by the National Renewable Energy Laboratory (NREL) in the United States. Gunturu and Schlosser (2011) reason that given the value of annual mean WPD of 300 W m^{-2} , which is the value used as a lower cutoff for the viability of commercial power generation, 200 W m^{-2} would be a reasonable cut-off for the instantaneous value. Also relevant in their rationale was the observation of very low power generation for typical wind turbines below a WPD of 200 W m^{-2} - typically, the power curve of a wind turbine increases very slowly up until about 200 W m^{-2} but rises quickly thereafter.

In addition to this, the estimation of the power that any individual wind turbine will produce must include numerous losses from availability (i.e. the wind turbine is stopped for reasons other than a lack of wind, e.g. down time for the maintenance of the turbine), electrical resistence and array interference when groups of turbines are clustered together. For example, electrical losses can be as high as 3% in large projects with long cable runs, and interference can cut production by up to 10% or more if the turbines are too close together, due to wake effects. When considered together, these losses can be significant; actual electricity delivery may be only 85-90% of a simple calculation of the potential power of a turbine (Gipe, 2004).

For our study we use an estimate of 3% of the rated power that the wind turbine itself uses for it's own operation and maintenance, which means that this 3% power is not available to an end user. The turbine has to generate at least that amount of power in order to 'break even' in terms of the net electricity generated. For a 1.5MW turbine, this corresponds approximately to 200 W m^{-2} .

Supplementary Information Figure Legends

Figure S1. An example of a histogram of wind power density that shows a typical skewed distribution.

Figure S2. Measures of variation. (a) the change in the RCoV from 50 m to 150 m, (b) the change in the IQR from 50 m to 150 m .

Figure S3. Measures of intermittency. (a) the change in the unavailability from 50 m to 150 m, (b) the change in the mean episode length from 50 m to 150 m.

Author contributions

W. Hallgren and U.B. contributed equally to this manuscript: W. Hallgren and U.B Gunturu generated the MERRA data, and wrote and edited the paper, W. Hallgren did most of the analysis in consultation primarily with U.B Gunturu and secondarily with C.A. Schlosser. U. B. Gunturu and C.A. Schlosser provided the prior analytical framework that this paper draws on with regard to the wind resource metrics used. C.A. Schlosser also provided editorial assistance.
